# Sublethal effects of acaricides and *Nosema ceranae* infection on immune related gene expression in honeybees

**DOI:** 10.1186/s13567-016-0335-z

**Published:** 2016-04-26

**Authors:** Paula Melisa Garrido, Martín Pablo Porrini, Karina Antúnez, Belén Branchiccela, Giselle María Astrid Martínez-Noël, Pablo Zunino, Graciela Salerno, Martín Javier Eguaras, Elena Ieno

**Affiliations:** Facultad de Ciencias Exactas y Naturales, Centro de Investigación en Abejas Sociales, Universidad Nacional de Mar del Plata–CONICET, Mar Del Plata, Buenos Aires Argentina; Departamento de Microbiología, Instituto de Investigaciones Biológicas Clemente Estable, Montevideo, Uruguay; Instituto de Investigaciones en Biodiversidad y Biotecnología (INBIOTEC-CONICET), CIB-FIBA, Mar Del Plata, Argentina; Highland Statistics, 03130 Alicante, Spain

## Abstract

*Nosema ceranae* is an obligate intracellular parasite and the etiologic agent of Nosemosis that affects honeybees. Beside the stress caused by this pathogen, honeybee colonies are exposed to pesticides under beekeeper intervention, such as acaricides to control *Varroa* mites. These compounds can accumulate at high concentrations in apicultural matrices. In this work, the effects of parasitosis/acaricide on genes involved in honeybee immunity and survival were evaluated. Nurse bees were infected with *N. ceranae* and/or were chronically treated with sublethal doses of coumaphos or *tau*-fluvalinate, the two most abundant pesticides recorded in productive hives. Our results demonstrate the following: (1) honeybee survival was not affected by any of the treatments; (2) parasite development was not altered by acaricide treatments; (3) coumaphos exposure decreased lysozyme expression; (4) *N. ceranae* reduced levels of vitellogenin transcripts independently of the presence of acaricides. However, combined effects among stressors on imagoes were not recorded. Sublethal doses of acaricides and their interaction with other ubiquitous parasites in colonies, extending the experimental time, are of particular interest in further research work.

**Electronic supplementary material:**

The online version of this article (doi:10.1186/s13567-016-0335-z) contains supplementary material, which is available to authorized users.

## Introduction

Pesticide exposure and pathogen infection are recognised as potential stressors affecting honeybee populations and consequently the profitability of beekeeping, such as parasitic mite syndrome or varroosis [1, 2], several viruses [3, 4], xenobiotics [5, 6] and microsporidiosis [7].

*Nosema ceranae* [8–10] is the predominant microsporidian pathogen, originally isolated from *Apis cerana* [11] but recently it has been reported as a natural parasite of *A. mellifera* over the world [12]. This obligate intracellular microparasite spreads among hosts via spores and is the etiologic agent of Nosemosis.

With respect to chemical stressors, honeybee colonies are exposed to pesticides used in agriculture or within bee hives by beekeeper intervention. In order to control *Varroa destructor*, the causative agent of varroosis, beekeepers apply synthetic acaricides, with the most commonly employed being the neurotoxic compounds coumaphos (organophosphate) and *tau*-fluvalinate (pyrethroid) (reviewed in [13]). These acaricides are applied directly to bee hives and accumulate in apicultural matrices, particularly beeswax [14], being detected in nearly all hives tested in several countries [15–19].

It has been suggested that *tau*-fluvalinate increases honeybee susceptibility to deformed wing virus infection [20], and that coumaphos can act as a systemic agent inside the colonies [21] and alters some metabolic responses [22]. Nevertheless, these molecules did not alter immunity at the gene expression level in bees subject to acute exposure in laboratory conditions [23].

Insects lack an acquired immune system but have a well-developed innate response [24]. In honeybees, humoral immunity involves antimicrobial peptide (AMP) synthesis [25]. Four AMP, abaecin, apidaecin, defensin and hymenoptaecin have been identified in *A. mellifera* when honeybees are challenged by bacteria [26, 27]. On the contrary, cellular defence involves phagocytosis, encapsulation and nodulation mediated mostly by hemocytes [28]. These latter processes are related to melanisation, a mechanism catalysed by phenoloxidase [29] and glucose dehydrogenase (GLD) [30]. Another component of worker bee defence is vitellogenin, synthesized by the fat body and released into the hemolymph [31, 32]. This yolk protein is involved in many physiological processes, for example in the regulation of honeybee longevity, immunity, brood food synthesis or acting as a stress response mediator [33]. Antúnez et al. [34] showed evidence that honeybee colonies with different degrees of *N. ceranae* infection could be associated to a differential expression of vitellogenin.

Currently, the requirement of high doses of acaricides due to *V. destructor* resistance added to the potential of honeybee colonies to accumulate acaricide residues and their metabolites has become detrimental to honeybee health and productivity. For this reason, the evaluation of sublethal effects of acaricides is essential to gain information on the long term effects of these widely used compounds in beekeeping.

The objective of this study was to determine whether exposure of adult bees to chronic sublethal acaricide (*tau*-fluvalinate and coumaphos) and/or infection with *N. ceranae* generates significant effects on individual immune components and honeybee survival.

## Materials and methods

### Colony health conditions and preparation of acaricide-free combs

Local hybrid honeybees (*A. mellifera mellifera*/*A. mellifera ligustica*) were obtained from colonies located in the experimental apiary J. J. Nágera coastal station (38°10′06″S, 57°38′10″W), Mar del Plata, Argentina.

Mite infestation levels in the apiary were monitored using the natural mite fall method [13]. Also, phoretic *Varroa* mite infestation rates were determined according to Fries et al. [35].

In order to evaluate the presence of *Nosema* spp. spores in the apiary, 60 forager honeybees returning from flight from each colony were macerated and examined using light microscopy [36]. Three colonies were selected to obtain brood combs based on the lowest prevalence recorded from *Varroa* mites (less than 1%) and the lowest abundance from *N. ceranae* (an average of 5 × 10^5^ *Nosema* spores/bee). Specifically, neither of the colonies used to obtain the imagoes presented any visible clinical symptoms of other diseases (i.e., American foulbrood or chalkbrood).

No chemical treatment was applied to the colonies 5 months before and during the experiment.

Four months prior to the beginning of the assays, in order to avoid the presence of long lasting acaricide residues in wax or honey [14], a plastic foundation was used allowing bees to draw out the foundation since new commercial beeswax foundation are paradoxically contaminated with residuals [37]. Plastic foundations were covered with a thin layer of virgin wax to get a better acceptance of these supports. Then they were placed in the selected colonies.

### *Nosema ceranae* spores

Foraging bees were collected from a naturally infected colony of the experimental apiary mentioned above. The spore number was determined using a hemocytometer under light microscopy. The presence of *N. ceranae* spores and the absence of *N. apis* spores was confirmed by PCR [38].

In order to avoid microbial contaminants and host tissue, fresh spore suspensions were purified following a triangulation method modified by Fries et al. [39]. The obtained suspension was immediately used for experimental infection.

### Chemicals

Technical grade chemicals were used for all trials. The acaricides coumaphos and *tau*-fluvalinate were obtained from Sigma-Aldrich^®^.

### Experimental infection with *N. ceranae* spores

Newly emerged bees, which are free from *N. ceranae* infection [9, 40], from three colonies were mixed together and were placed in wooden cages with a plastic mesh (11 × 9 × 6 cm^3^) in groups of 150. They were also provisioned with bee bread (stored at −20 °C for 3 months in order to eliminate viable spores) manually collected from plastic combs from honeybee colonies, water and sucrose syrup ad libitum.

Individual infection was achieved on day 3 after emergence according to Porrini et al. [41]. Honeybees were starved 5 h and then more than 600 individuals received 10 μL of sucrose–water solution (2:1 w:v) with 1.5 × 10^5^*Nosema* spores. The solution was continuously vortexed to ensure a uniform suspension. Approximately 650 control honeybees were treated in the same manner using sucrose-water solution (2:1 w:v) containing no *Nosema* spores. Those individuals that did not consume the total amount of solution were discarded from the assay. Then, they were caged in transparent and ventilated plastic flasks of 900 cm^3^, these hoarding cages have inputs for feeding devices and removable sides. Four replicates of 50 imagoes per treatment were made and maintained in an incubator (28 °C; 30% HR). Honeybees were fed ad libitum with bee bread, water and sucrose syrup. A chronology of the experiment is shown in Table 1.

**Table 1:** **Chronological description of experimental procedure**

1 day-old individuals	3 day-old	4 day-old	13 day-old
Cage rearing Diet: bee bread, sucrose solution (66%) and water ad libitum	Individual inoculation with or without *N. ceranae* spores in sucrose solution	Beginning of the administration of acaricide or sucrose only solution for controls Daily replacement of solutions until day 13 post emergence	Sampling bees for individual quantification of spores in midgut and individual gene expression analysis

### Selection of sublethal dose of acaricides

The experiment was designed in order to simulate realistic conditions of exposure to acaricides. Concentrations were based on previous data on the presence of these pesticides in pollen and honey. In the case of *tau*-fluvalinate, a concentration of 750 ppb was detected in honey [42] and 487.2 ppb in pollen [43], the selected dose was approximately the average between those values (666 ppb). In the case of coumaphos, 2020 ppb were found in honey samples [17] and 5800 ppb in pollen [18], so the selected dose was approximately the average (3333 ppb).

### Chronic exposure bioassays

Four-day-old worker bees were used for all bioassays (Table 1). Acaricidal compounds were dissolved in ethanol 96%. The final concentration of ethanol in sugar syrup was 2%; at this percentage it has been demonstrated that behaviour components are not affected in acute exposures [44]. Additionally, control bees received the same concentration of ethanol. Honeybees were treated *per os* with solutions of coumaphos (3333 ppb) or *tau*-fluvalinate (666 ppb) added to sugar syrup. Bees were supplied with one of the following diets: sucrose syrup + *tau*-fluvalinate, sucrose syrup + coumaphos or control syrup. Also frozen bee bread and water were administered ad libitum and honeybees were maintained in an incubator (28 °C; 30% HR) for 9 days under the treatments mentioned above. Solutions were administered in gravity feeders and were replaced daily, recording consumption every day through differences in feeder weight. Solution evaporation was checked in order to correct the consumed volumes.

In summary, honeybees were treated as follows: non-infected without acaricide exposure (C), non-infected exposed to *tau*-fluvalinate (CFluv), non-infected exposed to coumaphos (CCou), Infected without acaricide exposure (I), infected exposed to *tau*-fluvalinate (I + Fluv), infected exposed to coumaphos (I + Cou).

### *Nosema ceranae* development

Ten days post-infection, fifteen to thirty bees per replicate were analysed in order to individually quantify the parasite development. The digestive tract was removed by pinching the last abdominal segments and cutting the midgut. Midguts were stored at −20 °C until quantification. The number of spores in suspension (parasite intensity) was individually quantified with a hemocytometer under a light microscope [45].

### Bee sample preparation for molecular analysis

For total RNA extraction, ten bees belonging to each experimental group were collected at day 13 after emergence, sliced in pieces and individually merged in 1 mL of RNA later^®^ (Ambion, Inc. Austin, TX, USA) in order to avoid the degradation of mRNA.

### RNA isolation and cDNA synthesis

Ten bees per group (3 or 4 individuals per replicate) were individually analysed. RNA later was partially removed from samples. Honeybees were homogenised using a sterile glass rod and a sterile plastic tube. An aliquot of the body homogenates obtained to evaluate gene expression was also analysed to determine the presence of *Nosema* spores under light microscopy. Then, 600 µL of RLT buffer (Qiagen) were added to homogenates. Total RNA was isolated using the RNeasy plus mini kit (Qiagen), according to the manufacturer’s instructions. Total RNA recovered was immediately used to generate first strand cDNA using the quanti tect reverse transcription kit (Qiagen), according to the manufacturer’s instructions. Resultant cDNA synthesized were stored at −20 °C.

Negative controls were added in parallel for each step (RNA extraction and reverse transcription reactions).

### Gene and primer selection

In order to evaluate the effect of acaricides on immune-related genes of honeybees, transcript levels for the genes encoding the antimicrobial peptides abaecin (ABA), hymenoptaecin (HYM), defensin (DEF), the immune related proteins, glucose dehydrogenase (GLD), lysozyme (LYS), vitellogenin (VG) were assessed using primers previously described [25, 46, 47]. Gene transcripts were normalized relative to expression levels for the gene encoding ribosomal protein S5 (RPS5), a gene with constitutive expression across honeybee life stages [48] and disease status and β-actin, a moderately expressed housekeeping gene [47].

Primers used for quantitative PCR of honeybee transcripts are summarized in Additional file 1.

### Real-time PCR

Real-time PCR reactions for the amplification of specific genes were performed in 96-well plates using a BIO-Rad CFX-96 thermal cycler (Bio-Rad Corp.). Reaction mixtures consisted of 1× QuantiTect SYBR Green PCR Master Mix, 0.5 µM of each primer (one pair of primers per reaction), RNAse free water and 5 µL of 1:10 diluted cDNA in a final volume of 25 µL.

PCR reactions were conducted using a thermal program consisting of an initial activation step at 50 °C for 2 min and 95 °C for 15 min, and 39 cycles of 94 °C for 15 s, 50 °C for 30 s and 72 °C for 30 s. Fluorescence was measured in the elongation step. Negative controls lacking template were run by excluding nucleic acids from reactions.

Specificity of the reaction was checked by analysis of the melting curve of the final amplified product (from 65 to 95 °C).

### Normalization of the real-time data

Amplification results from different genes were expressed as the quantification cycle (Cq) value. According to a suggestion made by Vandesompele et al. [49], the geometric mean between the Cq of two reference genes (RPS5 and β-actin) was used for analysis. The expression ratio between each target gene and the geometric mean of reference genes was calculated according to the method described by Pfaffl [50].

### Statistical analyses

Prior to analysis, graphical explanatory techniques were applied to the original data to identify outliers and collinearity [51, 52].

#### Abundance of parasites

The abundance of parasites observed for each honeybee specimen was initially modelled using a generalized linear mixed model (GLMM) with a Poisson distribution and log link function. Nevertheless, due to more variability (overdispersion) than expected under the assumed distribution, a negative binomial distribution was preferred, resulting in the following model:

$${\text{Parasites}}_{\text{ij}} \sim {\text{NB }}(\mu_{\text{ij}} ,{\text{ k}});{\text{ Log }}(\mu_{\text{ij}} ) \, = {\text{ Intercept}} \, + \, {\text{ factor }}\left( {\text{Treatment}} \right)_{\text{ij}} \, + \, {\text{ a}}_{\text{i}} ,{\text{ where a}}_{\text{i}} \sim {\text{ N}}\left( {0, \, \sigma^{ 2}_{\text{cage}} } \right).$$

The random intercept a_i_ introduces a correlation structure between all observations from the same sampling cage [53, 54].

#### Survival analysis

A similar model was also performed using a GLMM with a binomial distribution. The response variable *Y*_*i*_ is the number of bees that survived after treatment, of *N*_*i*_ bees treated for a period of 13 consecutive days. To model this probability, we used the logistic link function of the form:

$${\text{logit}}\left( {\pi_{\text{ij}} } \right) \, = {\text{ Intercept }} + {\text{ factor }}\left( {\text{Treatment}} \right)_{\text{ij}} \, + {\text{ Days}}_{\text{ij}} \, + {\text{ a}}_{\text{i}} ,{\text{ where a}}_{\text{i}} \sim {\text{ N}}\left( {0, \, \sigma^{ 2} {\text{cage}}} \right).$$

The term a_i_ is a random intercept for cage_i_ and imposes a correlation on all observations from the same cage.

#### Consumption rates

The relationship between average food consumption (milligrams/hour/bee) versus day, treatment identity and their interaction, was defined using a linear mixed model (LME) [53, 55, 56]. As mentioned above, the term ai is the random effect and is assumed to be normally distributed with mean 0 and variances σ^2^. The term εij is the unexplained noise, and is assumed to be normally distributed with mean 0 and σ^2^.

The following model was applied:

$${\text{Food consumption}}_{\text{ij}} = {\text{ Intercept }} + {\text{ factor }}\left( {\text{Treatment}} \right)_{\text{ij}} *{\text{ Day}}_{\text{ij}} + {\text{ a}}_{\text{i}} + \, \varepsilon_{\text{ij}} .$$

#### Gene expression

Linear mixed effects models (LME) were used to model the gene expression as a response, in relation to the explanatory variable treatment. For each treatment level, gene expression was sampled from the same specimen. Hence, it is likely that individual gene expression is dependent on one another. Therefore to avoid a certain degree of pseudo-replication when fitting six separate models, an *N* by *N* correlation matrix was calculated a posteriori. The off-diagonal elements define the correlation between the residuals of each gene expression analysis and was used to assess dependency among the response variables. Thus, the following model was applied on the data obtained for each gene expression:

$${\text{Gene expression}}_{\text{ij}} = {\text{ Intercept }} + {\text{ factor }}\left( {\text{Treatment}} \right)_{\text{ij}} + {\text{ a}}_{\text{i}} + \, \varepsilon_{\text{ij}} .$$

The term a_i_ is the random effect representing the between-cage variation and is assumed to be normally distributed with mean 0 and variances σ^2^. The term ε_ij_ is the unexplained noise, and is assumed to be normally distributed with mean 0 and σ^2^. However, an initial analysis indicated large variation due to the effect of the covariate treatment, and therefore we allowed for heterogeneous residual variance structures [53, 55, 56].

The model selection followed the step-down approach described in West et al. [56] and Zuur et al. [53]. Significance of the variable treatment within the models was assessed using p values, which explains the effect of individual level of factor on the response variable as compared to the controls.

A detailed model validation was carried out by plotting residuals versus fitted values and versus each covariate to confirm that the underlying statistical assumptions were not violated [53, 54]. For the LME models, optimal model coefficients were derived using REML estimation [53, 56]. All analyses were performed with R (R Development Core Team, 2014) using the packages lme4 and nlme, respectively [57, 58].

## Results

### *Nosema ceranae* infection on honeybees

Sublethal doses of coumaphos and *tau*-fluvalinate did not alter *N. ceranae* development. Statistical analysis revealed no significant differences between experimental groups (X^2^ = 1.48, df = 2, *p* = 0.47).

The recorded mean of spores ± standard errors per bee in infected controls was 11 534 261 ± 1 236 776; 12 104 406 ± 1 081 263 for coumaphos treated bees and 11 923 054 ± 1 114 173 for *tau*-fluvalinate treated ones. Less than 8% of artificial infected honeybees did not have spores in their midgut when analysed by light microscopy.

No *N. ceranae* spores were recorded in non-infected honeybees, demonstrating that no spore contamination in the bee bread diet and the absence of unwanted infection of bees occurred during the assays.

### Honeybee survival

**Figure 1:**
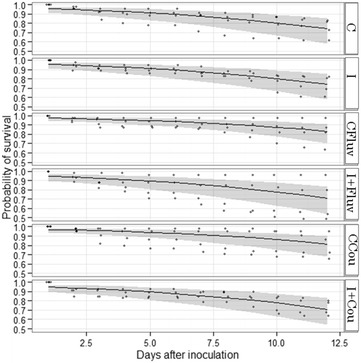
**Honey bee survival.** Predicted survival values according to each treatment and days after inoculation. Treatments: C (control); I (infected honeybees); CFluv (non-infected honeybees fed with 666 ppb of *tau*-fluvalinate); I + Fluv (infected honeybees fed with 666 ppb of *tau*-fluvalinate); CCou (non-infected honeybees fed with 3333 ppb of coumaphos); I + Cou (infected honeybees fed with 3333 ppb of coumaphos).

Model selection indicated that there was no significant effect of treatment identity on survival rates (X^2^ = 3.29, df = 5, *p* = 0.65) indicating that administrated coumaphos and *tau*-fluvalinate did not alter mortality of infected or non-infected honeybees. However, the probability of survival declined significantly after the start of the experiment (X^2^ = 534.14, df = 1, *p* < 0.001) (Figure 1).

### Food intake

The optimal model for food consumption was an LME, where food consumption was mainly affected by treatment variable (L-ratio = 22.23, df = 5, *p* < 0.001). There was no significant interaction between the days of consumption and treatments (L-ratio = 4.13, df = 5, *p* = 0.53). The effect of time (days) was neither significant (L-ratio = 0.86, df = 1, *p* = 0.37).

**Figure 2:**
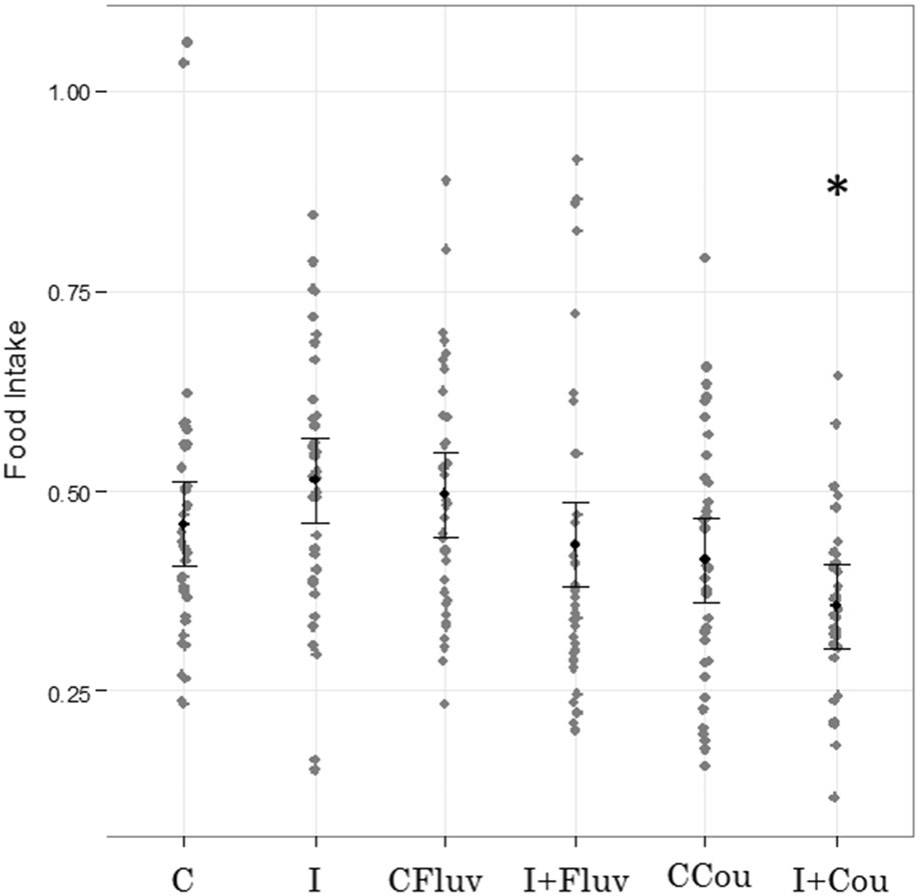
**LME results for food consumption.** Sucrose consumption is expressed as the amount of sucrose solution (50% w/v, ad libitum delivery) consumed daily, per hour and per bee during the assay (food intake). The black dots represent the fitted values, and the lines are 95% confident intervals. Raw data were superimposed (grey dots). Treatments: C (control); I (infected honeybees); CFluv (non-infected honeybees fed with 666 ppb of *tau*-fluvalinate); I + Fluv (infected honeybees fed with 666 ppb of *tau*-fluvalinate); CCou (non-infected honeybees fed with 3333 ppb of coumaphos); I + Cou (infected honeybees fed with 3333 ppb of coumaphos). Asterisk indicates significant differences (df: 207; *p* = 0.0078) with control treatment (C).

Significant differences were only detected on infected honeybees that received coumaphos treatment when compared to controls (*p* = 0.007). Figure 2 shows the results of LME for food consumption.

### Immune-related gene expression

Expression levels of genes involved in immune response were determined 10 days after infection (13 days after emergence). Gene-specific amplification was confirmed for the six genes analysed, as a single peak in the melting curve analysis and through the Tm values. No amplification occurred for negative controls (non-template controls).

*Nosema ceranae* spore presence was confirmed, under a light microscope, before total RNA extraction in all infected honeybee samples. In contrast, no spores were detected in non-infected honeybees.

Two of the six statistical models did not fulfil the assumptions of the LME and therefore we failed to validate them. This was mainly due to the presence of negative fitted values and/or heterogeneity of variances. Therefore no results are presented for hymenoptaecin and glucose-dehydrogenase gene expressions. With respect to the defensin gene expression, the data set contained a considerable number of missing values for a level of the factor treatment, therefore we could not include the “non-infected exposed to coumaphos” level in the analysis.

### Genes that encode for antimicrobial peptides

Treatments did not show a significant effect on abaecin (L-ratio = 9.48, df = 5, *p* = 0.09) nor defensin transcript levels (L-ratio = 0.85, df = 5, *p* = 0.97).

### Genes that encode lysozyme and vitellogenin

**Figure 3:**
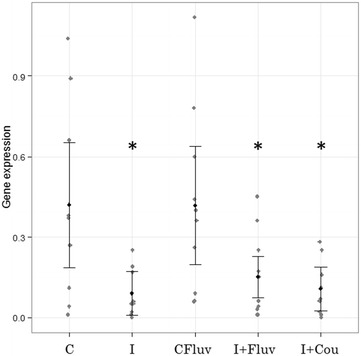
**Vitellogenin mRNA expression.** LME results. The black dots represent the fitted values, and the lines are 95% confidence intervals. Raw data were superimposed (grey dots). Gene expression was primarily affected by treatment (L-ratio = 12.27, df = 4, *p* = 0.01). Treatments: C (control); I (infected honeybees); CFluv (non-infected honeybees fed with 666 ppb of tau-fluvalinate); I + Fluv (infected honeybees fed with 666 ppb of *tau*-fluvalinate); I + Cou (infected honeybees fed with 3333 ppb of coumaphos). Treatments I, I + Fluv and I + Cou were significantly lower than control treatment (t = −2.62, t = −2.13 and t = −2.47 respectively; *p* = 0.01, *p* = 0.04 and *p* = 0.02 respectively). Asterisk indicates statistically significant differences with treatment (C).

The optimal model describing vitellogenin mRNA levels was an LME that allowed for unequal variances depending on infection vs non-infection. The “non-infected exposed to coumaphos” treatment was removed from the statistical analysis due to a considerable number of missing values that were not accepted by the model. Vitellogenin gene expression was affected by treatments (L-ratio = 12.27, df = 4, *p* = 0.01). This is a significant, albeit weak effect (Figure 3).

*Nosema ceranae* infection caused a reduction in transcript abundances of vitellogenin (VG) when compared to the control treatment (*p* = 0.01). Also, when both stressors were applied a drop of VG was detected (*p* = 0.02).

The *tau*-fluvalinate treatment did not cause a reduction by itself in VG gene expression; however the combination of this acaricide and *N. ceranae* infection did (*p* = 0.04) (Figure 3).

**Figure 4:**
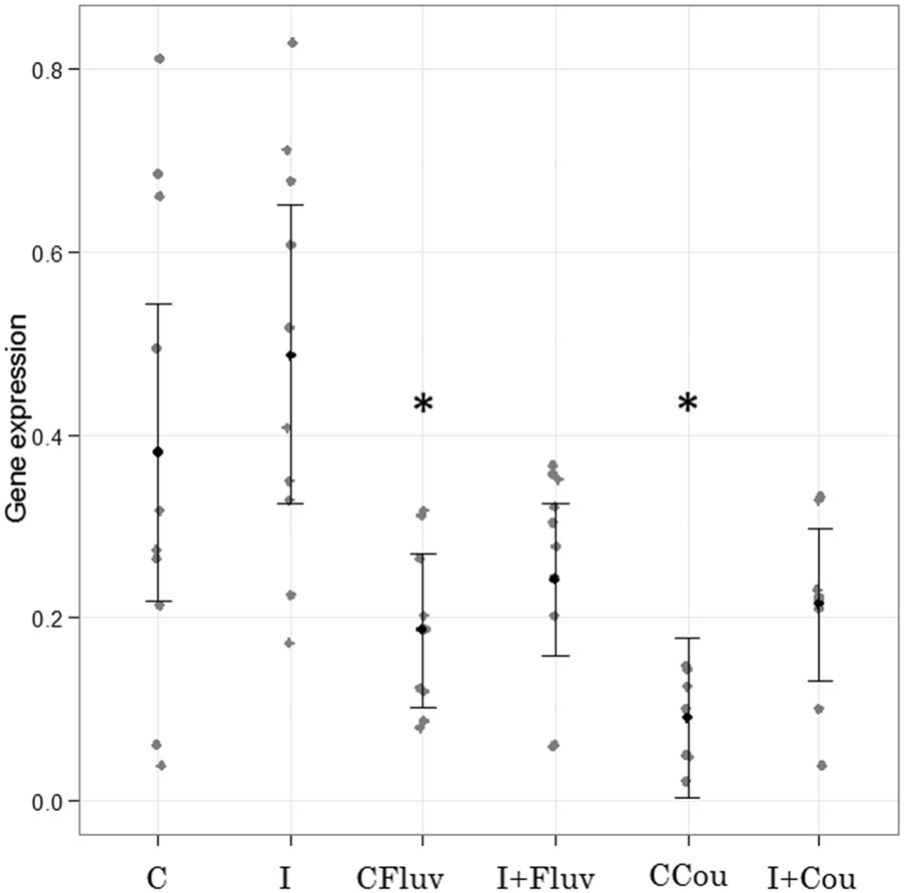
**Lysozyme mRNA expression.** Results of the LME. The black dots represent the fitted values, and the lines are 95% confidence intervals. Raw data were superimposed (grey dots). Gene expression was primarily affected by treatment (L-ratio = 20.92, df = 5, *p* < 0.001). Treatments: C (control); I (infected honeybees); CFluv (non-infected honeybees fed with 666 ppb of *tau*-fluvalinate); I + Fluv (infected honeybees fed with 666 ppb of *tau*-fluvalinate); CCou (non-infected honeybees fed with 3333 ppb of coumaphos); I + Cou (infected honeybees fed with 3333 ppb of coumaphos). When compared with control, treatment CCou was significantly lower (t = −3.09; *p* < 0.01) and treatment CFluv also had a negative but weak influence on the gene expression (t = −2.09; *p* = 0.05). The asterisk indicates statistically significant differences with respect to control treatment (C).

In the case of lysozyme expression analysis, the optimal model was also an LME with unequal variances. The results revealed that lysozyme transcript levels were primarily affected by treatment (L-ratio = 20.92, df = 5, *p* < 0.001). When comparing the treatment levels with the control, we found that non-infected honeybees exposed to coumaphos expressed significantly lower relative abundances of transcript compared to the baseline (t = −3.09; *p* < 0.01), and that non-infected honeybees that received *tau*-fluvalinate treatment also had a negative but weak influence on gene expression (t = −2.09; *p* = 0.05) (Figure 4).

## Discussion

In spite of the ubiquitous presence of *N. ceranae* and highly persistent acaricides in apicultural matrices, our results revealed under carefully controlled laboratory conditions, no evidence of combined effects of those stressors on honeybee survival and in most of the immune related genes analysed.

It is worth noting that coumaphos and *tau*-fluvalinate concentrations provided in our study have been reported in contaminated honey and pollen stored inside hives [17, 18, 45, 46], therefore acaricide-honeybee interactions are likely to occur under field-realistic scenarios. Chronic administration of these acaricides during 9 days did not affect worker survival, nor in coexposure with *N. ceranae* under laboratory conditions. Our test bees only received acaricide exposure as imagoes, and larvae developed in a reduced acaricide environment, therefore it will be interesting to analyse exposure to sublethal levels on survival even during larval development.

It is unsurprising that insecticides alter insect behaviour, such as feeding levels but, in this work, intake of diets was similar to control group. In other words, the observed responses were not due to poor nutrition. Only infected bees that received syrup contaminated with coumaphos showed a reduced consume when compared to controls. This result might be clarified by performing long lasting assays.

Although colonies employed during the assays did not show any clinical symptom of diseases, pathogens could be present in the administered inoculum or in the pollen diet. However, until this work was conceived, no published method have been proposed in order to avoid the possible presence of pathogens without altering the highest nutritional quality of beebread. This should be considered in future experimental designs.

Recently, an increase in *Nosema* spore loads in colonies treated with the insecticide imidacloprid was shown, suggesting an indirect effect of pesticides on pathogen growth in honeybees [59]. In our study, *Nosema* growth rate did not change in the presence of coumaphos or *tau*-fluvalinate at sublethal doses, in agreement with recent field work in naturally infected colonies [60].

*Nosema ceranae* infection by itself did not have an effect on genes that encode antimicrobial peptides nor tested enzymes at day 10 post infection. Only a significant downregulation of vitellogenin was detected in *N. ceranae* infected honeybees. These results agreed with those found by Antúnez et al. [61] who showed that vitellogenin transcripts decreased in *N. ceranae* infected bees at 7 days post infection. However, Chaimanee et al. [62] did not find differences in vitellogenin expression levels at day 13 post emergence but methodologies were dissimilar, mainly since they did not provide any protein source. In reference to the importance of the diet, a positive correlation between the level of pollen consumption and Vg expression in *Nosema*-infected bees has been widely demonstrated, underlining the importance of the diet [63].

Although a reduction in Vg transcripts was recorded in infected honeybees treated with *tau*-fluvalinate and coumaphos, this was due to the parasite action. The vitellogenin protein is a central regulator of lifespan, supporting hemocyte viability [32], recognizing cell damage [64] and acting as a metabolic regulator of stress responses [33]; altering its expression rates induced by the presence of *N. ceranae*, independently of acaricide exposure, could lead to an acceleration on honeybee ontogeny [33] and reduction of its antioxidant capacity. The limited knowledge of the impact of chemical pesticides on humoral immunity postulates that they do not appear to affect AMP production [65]. This agrees with our study, since no differences in defensin or abaecin transcript levels were found on bees exposed to *tau*-fluvalinate or coumaphos treatments.

In the present study, effects on the lysozyme gene on honeybees at sublethal chronic exposure were recorded but in a previous report, no change was observed under acute exposure (LC_50_) employing the same acaricides [23]. Although it should be considered that different methodologies related to acaricide exposure were employed, such as the evaluation of acute toxicity by contact, we can hypothesize that huge amounts of these molecules administrated over 24 h do not impact on the immune system as much as little doses over a longer period of time; it seems that chronic stress can lead to a dysregulation of the physiological pathways to compensate for the stress response.

The common feature of lysozymes in animals is their ability to hydrolyse bacterial cell wall [66] and to promote the synthesis of other antimicrobial peptides [67]. Chronic exposure to coumaphos reduced lysozyme transcripts but in exposed honeybees infected with *N. ceranae* no differences were observed with respect to control treatments. This result may be due to the absence of response by individual honeybees in the presence of both stressors or that the parasite induces an up-regulation of this gene while the acaricide downregulates it, buffering the net effect. The energetic cost could be the key reason behind this result because this parasite generates an energetic demand on its host. This could affect the mechanisms that regulate the mobilisation of energy reserves in infected individuals [68, 69].

The long term effects of *tau*-fluvalinate and coumaphos and their interaction with other ubiquitous parasites are of particular concern. Thus, the possible detrimental effects of these stressors on colony health and performance cannot be discarded and warrant further research. Our interest in future studies will be to intensify sampling in order to reduce variability between individuals and to perform again those treatments or response variables that were necessary to exclude from statistical analyses. Also, since honeybee physiology is characterized by a surprising plasticity and is regulated by colony signals and resources, further field or semi-field studies are necessary.

## Additional file

10.1186/s13567-016-0335-z **Sequences of oligonucleotide primers used for real-time PCR quantification.** Table provides primer sequences, melting temperatures and reaction efficiencies.
