# Supplementary material for: Sublethal effects of acaricides and Nosema ceranae infection on immune related gene expression in honeybees
Source: Vet Res. 2016 Apr 26;47:51. doi: 10.1186/s13567-016-0335-z (PMC4847213; doi:10.1186/s13567-016-0335-z)
Supplement: Supplementary file 1 — 10.1186/s13567-016-0335-z Sequences of oligonucleotide primers used for real-time PCR quantification. Table provides primer sequences, melting temperatures and reaction efficiencies. [file 13567_2016_335_MOESM1_ESM.doc]

| Amplification target | **Primer** | **Sequence** | Tm (°C) | Efficiency | Reference |
| --- | --- | --- | --- | --- | --- |
| Ribosomal protein S5 | RPS5-F | 5’-AATTATTTGGTCGCTGGAATTG-3’ | 75.8 | 2.05 | [26] |
|  | RPS5-R | 5’-TAACGTCCAGCAGAATGTGGTA-3’ |  |  |  |
| Antimicrobial peptide abaecin | Abaecin- F | 5’-CAGCATTCGCATACGTACCA-3’ | 78 | 2.06 | [26] |
|  | Abaecin- R | 5’-GACCAGGAAACGTTGGAAAC-3’ |  |  |  |
| Antimicrobial peptide defensin | Defensin- F | 5’- TGTCGGCCTTCTCTTCATGG-3’ | 78.3 | 2.07 | [50] |
|  | Defensin- R | 5’- TGACCTCCAGCTTTACCCAAA-3’ |  |  |  |
| Antimicrobial peptide hymenoptaecin | Hymenopt- F | 5’-CTCTTCTGTGCCGTTGCATA-3’ | 80.5 | 2.03 | [26] |
|  | Hymenopt- R | 5’-GCGTCTCCTGTCATTCCATT-3’ |  |  |  |
| Vitellogenin | VgMC-F | 5’-AGTTCCGACCGACGACGA-3’ | 80.3 | 2.17 | [49] |
|  | VgMC-R | 5’- TTCCCTCCCACGGAGTCC- 3’ |  |  |  |
| Glucose dehydrogenase | GLD- F | 5’- CTGCACAACCACGTCTCGTT-3’ | 86.5 | 2.22 | [50] |
|  | GLD- R | 5’- ACCGCCGAAGAAGATTTGG-3’ |  |  |  |
| Lysozyme | LYS- F | 5’- ACACGGTTGGTCACTGGTCC-3’ | 82.5 | 2.03 | [50] |
|  | LYS- R | 5’- GTCCCACGCTTTGAATCCCT-3’ |  |  |  |
| β-actin | BACT- F | 5’- ATGCCAACACTGTCCTTTCTGG-3’ |  | 1.99 | [50] |
|  | BACT- R | 5’- GACCCACCAATCCATACGGA-3’ |  |  |  |
